# Alteration of enzyme activities and *MYb10* gene expression in response to brown rot disease in two apple cultivars

**DOI:** 10.1016/j.bbrep.2025.102166

**Published:** 2025-07-17

**Authors:** Mohammad Reza Raji, Sepideh Sanjari, Fatemeh Derikvand, Mitra Khademi, Mostafa Farajpour

**Affiliations:** a Department of Horticultural Science, Faculty of Agriculture, Lorestan University, Khorramabad, Iran; b Department of Agronomy and Plant Breeding, College of Abourihan, University of Tehran, Tehran, Iran; c Department of Plant Protection, Faculty of Agriculture, Lorestan University, Khorramabbad, Iran; d Department of Plant Production and Genetic Engineering, Faculty of Agriculture, Lorestan University, Khorramabad, Iran; e Crop and Horticultural Science Research Department, Mazandaran Agricultural and Natural Resources Research and Education Center, Agricultural Research, Education and Extension Organization (AREEO), Sari, Iran

**Keywords:** Disease resistance, *MdMYB10*, *Monilinia laxa*, Post-harvesting, Defense enzymes

## Abstract

Apples (*Malus domestica*) are widely enjoyed but are prone to fungal infections, notably brown rot caused by Monilinia spp., which significantly impacts postharvest quality. This study evaluated the activities of catalase (CAT), peroxidase (POD), phenylalanine ammonia lyase (PAL), and polyphenol oxidase (PPO), along with the expression of *MdMYB10* in two apple cultivars (Gala Imperial and Lebanese) after inoculation with *Monilinia laxa* over 1, 2, and 7 days. Results showed that enzyme activities and *MdMYB10* expression significantly increased post-infection, indicating their roles in the defense against brown rot. Notably, a strong positive correlation was found between *MdMYB10* upregulation and the activities of PAL, POD, and PPO. PAL activity peaked at 2 days post-inoculation, while *MdMYB10* expression, along with POD and PPO activities, reached their highest levels by day 7. These findings suggest that *MdMYB10* expression enhances the activities of these defense enzymes, with PAL initiating the response by producing phenolic compounds that serve as substrates for PPO and POD, aiding in disease resistance. Although PAL, POD, and PPO activities increased after *M. laxa* inoculation, no significant differences in PPO and POD activities were observed between cultivars, while Gala Imperial displayed higher PAL activity, enhancing its resistance to infection. The study highlights *MdMYB10* as a potential candidate for breeding programs aimed at improving brown rot resistance in apples.

## Highlights

• Upregulation of MdMYB10 enhances defense enzyme activities (PAL, POD, PPO) against brown rot.
• Time-dependent increase in CAT, POD, PAL, and PPO activities post-inoculation with *Monilinia laxa.*
• Gala Imperial shows higher PAL activity, indicating better resistance to *M. laxa* than Lebanese apples.
• MdMYB10 is a promising target for developing disease-resistant apple cultivars through biotechnology.

## 1 Introduction

Apple (*Malus domestica*) from the Rosaceae family was domesticated more than 8000 years ago and has become one of the most popular fruit trees in the world [1]. More than 7500 apple cultivars are cultivated in different parts of the world because of their well adaptation to various climates, and worldwide apple production is around 93 million tons [2]. In recent years, apple production has increased substantially due to favorable weather conditions in Iran, where it ranks fourth in apple production in the world with 2.24 million tons annually [3,4]. Apples contain high amounts of polyphenols, diverse phenolics such as flavonoids and phenolic acids in their peel and flesh, which are beneficial for human health and help to prevent various diseases by exhibiting antioxidant activity [5]. The amounts of these biochemical components in apple varieties exhibit significant differences [6].

Viral, fungal, and bacterial pathogens are able to infect fruit in orchards and also during post-harvesting, which causes a reduction in the quantity and quality of production [7]. Among fungal rot diseases, brown rot caused by Monilinia spp. (*M. laxa*, *M. fructigena*, and *M. fructicola*) is a worldwide scourge that severely damages stone and seeded fruit trees in orchards and also infects fresh fruit after harvesting [8,9]. Brown rot causes blossom blight, twig cankers, and fruit rot in orchards and spreads in fruit storage by transmitting infected fruits or fruit surface fungal contamination [[10], [11], [12]]. The colony color and size of *M. laxa* and *M. fructigena* are greenish-gray with an average size of less than 0.5 mm and white to light beige with an average of 1.5 mm, respectively [13]. *M. laxa* and *M. fructigena* are the most important fungal pathogens responsible for producing rounded brownish spots in the central infected site on apples, which dramatically reduces apple production every year in pre- and post-harvest stages in Iran and the world [13,14].

When plants are exposed to pathogen infection, active oxygen forms are highly generated, leading to membrane lipid peroxidation and rigidification, protein denaturation, DNA mutation, leaf senescence, flower wilting, fruit ripening, and postharvest fruit spoilage. Therefore, detoxification of tissues from them is necessary during the stresses, which is done by enzymatic and non-enzymatic components. The main antioxidant enzymes are catalase (CAT), superoxide dismutase (SOD), ascorbate peroxidase (APX), peroxidase (POD), glutathione peroxidase, and reductase (GPX and GR) [15].

Moreover, phenolic compounds play a role in the protection of plants against environmental stresses and are synthesized in response to stresses [16]. They are involved in antioxidant activity to scavenge free radicals, metal detoxification, protection against lipid peroxidation, and the lignification process [[17], [18], [19]]. Most of the phenolic compounds are the products of branches of the phenylpropanoid pathway and have higher antioxidant activities than non-enzymatic antioxidant components such as carotenoids and vitamins C [20,21]. The first step of the phenylpropanoid pathway is activated by phenylalanine ammonia lyase (PAL) enzyme [21]. PAL plays a role as an antioxidant enzyme by capturing reactive oxygen species (ROS) via the synthesis of phenolic compounds, and its activity increase is sometimes used as a biochemical marker for the resistance of plants to stress [22]. Another enzyme that plays a vital role in defense against pathogen attacks is polyphenol oxidase (PPO). Although PPO, as a browning enzyme, is not a primary component of the antioxidant defense mechanism, it is involved in the oxidation of phenolic compounds to quinones, which have an extremely toxic effect on pathogens, thereby improving fruit disease resistance [20,23].

It is noteworthy that environmental stress is first sensed by plants, and then specific signals with regulating transcription factors (TFs) are generated in the defense system. MYBs are one of the fourth important TF families that play a role in response to biotic and abiotic stress and are also involved in development and metabolite biosynthesis such as anthocyanin and flavonol [24]. For example, the *MYB10* gene in apple is allelic to *MYB1*/*MYBA* of Arabidopsis, regulates anthocyanin biosynthesis, and its over-expression leads to an enhancement of anthocyanins in whole fruit 20-fold more than their levels in non-transgenic samples [25]. Moreover, ethylene biosynthesis in transgenic apple with *MYB10* over-expression was enhanced through the ethylene-related TFs, and premature ethylene production caused a reduction in fruit quality [1].

Due to the brown rot caused by *Monilinia laxa*, significant post-harvest losses in apples, and the disruption of ROS metabolism that accelerates fruit disease development, enhancing antioxidant systems and synthesizing plant secondary metabolites (PSMs) can improve resistance to post-harvest diseases [[26], [27], [28]]. In light of the substantial climate change affecting Iran, which results in decreased yields, it is increasingly crucial to sustain current crop productivity [29]. Therefore, the aim of the current study was to 1) assess the activities of antioxidant (CAT) and browning enzymes (PAL, PPO, and POD) in apple fruit of two cultivars (Gala Imperial and Lebanese) after 1, 2, and 7 days of inoculation with the *M. laxa* pathogen, and 2) analyze the expression pattern of the *MYB10* gene in response to this fungal infection to determine its role in fruit resistance to brown rot disease.

## 2 Material and method

### 2.1 Herbal materials

In this study, fresh fruits of Lebanese and Gala Imperial cultivars were harvested from an orchard located in Khorram Abad, Lorestan, Iran. The apples were selected based on their uniformity in size, weighing approximately 190 ± 5 g.

### 2.2 Inoculation of apples by *Monilinia laxa* phytoene

To validate the occurrence of pathogenicity by the apple brown rot fungus, a method previously described by Etebarian et al. [30] was employed. A spore suspension was prepared at a concentration of 106 spores per ml from pathogenic isolates using a hemacytometer. Three infected apples from each cultivar were included in each treatment group. The apples were disinfected with a 2 % sodium hypochlorite solution for 2 min, washed several times with distilled water, and then immersed in 90 % ethyl alcohol for 5 s. A sterile needle was used to create a 0.5 mm diameter and 3 mm deep hole in each apple at equal distances. Next, 20 μl of the *Monilinia laxa* suspension was injected into each hole. The apples were then placed in disposable plastic containers and each container was placed in a freezer bag. Infected Apples after 1, 2 and 7 days of inoculation as well as control were sampled for assessing enzymes activities and RNA extraction.

### 2.3 Assessment of apple enzyme activities in response to *Monilinia laxa* attack

The activities of CAT and POD enzymes were measured following the method described by Derikvand et al. [31]. Briefly, 2 g of each samples were collected in a mortar and cold extraction solution containing 0.8 M potassium chloride (KCl) and a 10 M sodium phosphate buffer (pH 7) was added and ground well. The mixture was then centrifuged at 12,000 revolutions per minute for 20 min at a temperature of 4 °C. The supernatant served as the source for enzyme activity assays using ultraviolet spectrophotometry. POD activity was assessed by measuring the oxidation of guaiacol. POD and CAT were defined as the amount of enzyme that results in a 0.1 increase of absorbance at 470 and 240 nm per minute, respectively.

To assess the PPO and PAL activities, 2 g of each sample was ground with 10 ml of a cooled buffer (50 mmol l^−1^) containing 1.33 mmol l^−1^ EDTA and 1 % PVPP in mortar, then samples were centrifuged at 12,000×*g* at 4 °C for 15 min. The supernatant was collected and used as the crude enzyme solution for further analyses. The activities of PPO and PAL were determined according to the method described by Apaliya et al. [32], with some modifications. One unit of PPO and PAL activity was defined as an increase in absorbance of 0.01 per minute at 398 nm and 290 nm, respectively. All enzyme activities were expressed as U g^−1^ FW (fresh weight).

### 2.4 RNA extraction and *MdMYB10* expression analysis in response to brown rot disease

Total RNA was extracted from samples using an RNA extraction kit (RNXplus) following the manufacturer's instructions (CinnaGen, Iran). The quality and quantity of the extracted RNA was determined by agarose gel electrophoresis and spectrophotometer, respectively. After removing the possible DNA contamination with DNAse I, the cDNAs were synthesized from DNA-free total RNAs of each sample using the RevertAid Reverse Transcriptase kit (Thermo Scientific Company, Germany) based on the manufacturer's manual. Quantitative real-time PCR was carried out in three independent biological replicates using a MiniOpticon qPCR detection system (Bio-rad) and the SYBR Green super-mix solution (CinnaGen, Iran) according to the instructions protocol. The amplification program was performed under the following conditions: initial denaturation at 94 °C (5 min), 40 cycles at 94 °C (30 s), 60 °C (30 s) and 72 °C (30 min) for denaturation, annealing and extension, respectively. The actin gene and *MdMYB10* gene expression in each cultivar under control condition (non-infected apple) were used as an internal control and calibrator, respectively. Relative gene expression levels were calculated by the 2^−^^ΔΔCT^ method [33], and specific forward and reverse primers for *MdMYB10* amplifying were (F: 5′GGAGGGGAATGAAGAAGAGG3′) and (R: 5′TCCACAGAAGCAAACACTGAC3′), respectively. In addition, the actin primer (F: 5′-GGAGAAGATTTGGCATCATCACTTTCTACAATGAG-3′) and (R: 5′-CTTCCTGATATCCACAATCACATTCATGATGC-3′) were used as internal control.

### 2.5 Statistical analysis

A one-way ANOVA was conducted on data and means were compared by the least significant difference (LSD) test at significance level of 5 % (p < 0.05) using SAS software [34].

Also, Pearson's correlation analysis was performed to evaluate the relationship between data and related heat map was visualized by MetaboAnalyst.

## 3 Result and discussion

### 3.1 Changing enzyme activities in response to *Monilinia laxa* infection in apples

Brown rot is the one of most important fungal disease of apples caused by *Monilinia* spp., which leads heavy losses predominantly in postharvest [35]. Conidia of Monilinia spp. are produced during the growing season and can infect apples at any developmental stage of fruits but brown rot disease is observed during the post harvesting stage largely linked to the infection occurring just before harvesting [35]. Plant response to pathogen attacks involves the synthesis and accumulation of plant secondary metabolites (PSMs) [28] and induction of the fruit resistance to postharvest diseases is associated with defense enzymes [26,27].

Antioxidant enzymes such as CAT and POD are involved in defense mechanism of plants which protect cells by mitigating active oxygen damage [36].

**Fig. 1:**
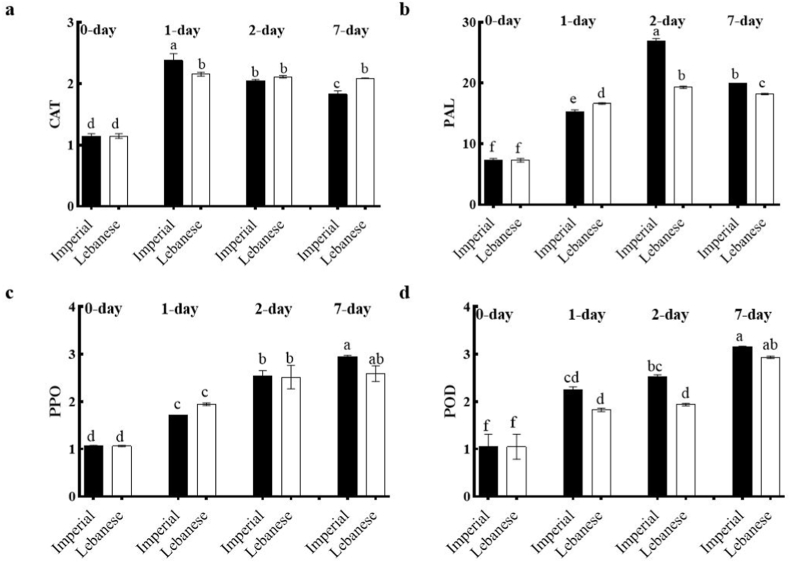
Alteration of enzymes' activities in fruits of two apple cultivar after 1, 2 and 7 days of *M. laxa* infection. All enzyme activities were expressed as U g^−1^ FW (fresh weight). The same letter indicates no significant difference at the 5 % level of probability. Each value represents the average of three replications, with five samples in each replication, totaling 15 samples.

Our results showed that CAT activity increased after a day of *M. laxa* inoculation in both cultivars, but its level decreased and did not change after 2 and 7 days of inoculation in Imperial and Lebanese, respectively (Fig. 1a). Previous study reported that the level of hydrogen peroxide (H_2_O_2)_ increased after apples infected by *M. laxa* and increment of antioxidant enzymes such as CAT and POD improved the apple defense system. Moreover, they reported that CAT activity was increased after apple infection by *M. laxa* and then decreased which was in line with our results [31].

PAL, PPO and POD not only play roles in enzymatic browning phenomena of crops but also are directly involved in resistance to pathogenic agents [37]. PAL is the first enzyme of phenylpropanoid metabolism pathway, thus playing a vital role in the synthesis of defense phenolic compounds such as phenols and lignin. Moreover, it has antioxidant enzymatic roles during the synthesis of phenolic compounds by capturing the ROS [22,38,39]. PPO and POD are involved in the oxidation of phenolic compounds and participating in hormone synthesis, so fruit disease resistance is enhanced [40]. POD prevents the cellular spread of infection by creating structural barriers or generating toxic environments [37].

Also, PPO is involved in resistance to biotic stress via the three mechanisms which are 1) catalyzing the phenol oxidation to quinones, which are extremely harmful to pathogens, 2) generating a physical barrier by producing melanin around injured tissue through the cross-linking between quinones with proteins or other phenols, and 3) modifying proteins leading to alkylation, which prevents nutrient absorption in microorganisms [23,41]. The results showed that POD, PPO and PAL enzymes activities were significantly increased after *M. laxa* infection in both apple cultivars (Imperial and Lebanese). After inoculation, PAL activity increased significantly, peaked on day 2 and then decreased on day 7, with significant differences in cultivars at all-time points (Fig. 1b).

PPO activity significantly enhanced with increasing time of inoculation from 1 to 7 days in both cultivars, but its level showed no significant differences in cultivars (Fig. 1c). Although POD activity increased after apples were infected by the pathogen and reached its highest level on day 7 in both cultivars, a significant difference between cultivars was observed only on 2 days after inoculation (Fig. 1d). Based on the aforementioned results, it seems that these three enzymes are involved in the apple defense mechanism against *M. laxa* attack.

**Fig. 2:**
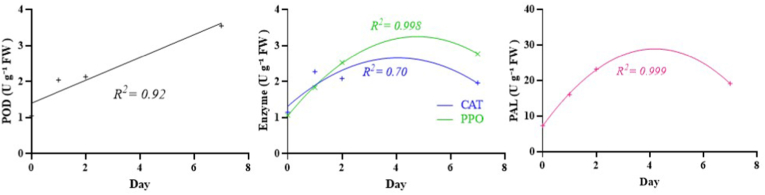
Linear (POD) and quadratic (CAT, PPO, and PAL) models of the four enzymes across the infection timeline in apple.

To statistically validate the temporal dynamics of enzyme activities, we applied linear and quadratic regression analyses to the averaged data from both cultivars (Imperial and Lebanese) across the infection timeline (0, 1, 2, and 7 days). For POD, a significant linear regression model was observed (R^2^ = 0.92), indicating a steady, time-dependent increase in activity (Fig. 2). In contrast, CAT, PPO, and PAL exhibited non-linear patterns, where quadratic regression provided superior fits (R^2^ = 0.70 for CAT, R^2^ = 0.998 for PPO, and R^2^ = 0.999 for PAL). The pronounced quadratic trajectories suggest these enzymes follow a biphasic response—characterized by an initial surge and subsequent decline (or stabilization)—during infection.

Interestingly, the highest level of PAL activity was observed 2 days after inoculation, but the highest level of POD and PPO was reached on day 7. So, it could be hypothesized that PAL enzyme is activated before PPO and POD in apples infected by *M. laxa* due to the its responsibility in producing phenolic compounds, which PPO and POD use as substrates in oxidative processes. Also, based on the observed activity pattern of the aforementioned enzymes and their significant differences in activity between cultivars, it seems that PAL is more important than the other two enzymes in the defense mechanism.

### *3.2* *MYB10* expression analysis and predicts its role in response to *Monilinia laxa* infection in apples

Biosynthesis and accumulation of plant secondary metabolites (PSMs) are controlled by genetic factors such as transcription factors (TFs) and genes [28]. TFs are a group of DNA-binding proteins that are involved in gene expression regulation during plant development and defense system [42,43]. Four families of TFs, including MYB, NAC, WRKY and AP2/ERF, are regulators of gene expression that play a vital role in response to various stresses, making them good candidates for genetic engineering to improve plant immunity against environmental stresses [44,45].

MYB (Myeloblastosis) TFs are one of the largest TF **families** in plants, which are involved in developmental processes, hormone signaling, and regulation of secondary metabolite biosynthesis [24]. Each member contains one to four repeats of the MYB domain (a conserved DNA-binding site), and based on these **repetitions** and the **position** of the domains, they are divided into different classes including 1R-MYB (R1/2, R3), 2R-MYB (R2R3), 3R-MYB (R1R2R3), and 4R-MYB (R1/R2-like repeats) [46]. Genes of two-repeat (R2R3) MYB TFs have been isolated from many monocots and dicots, and **these genes play a role** in regulating **metabolism** such as anthocyanin biosynthesis, disease resistance, morphogenesis, and cell differentiation in plants [47]. Interaction among Basic helix-loop-helix (bHLH), WD40, and R2R3 MYB transcription factors is essential for regulating anthocyanin biosynthesis in plants [48,49].

*MdMYB10*, from the R2R3 MYB subfamily in apple, **has** 58 % protein similarity to PAP1/MYB75 in Arabidopsis and regulates anthocyanin biosynthesis, contributing to red flesh color by interacting with **MdbHLH3/33** to enhance **MdDFR** promoter activity [1,50]. The *MdMYB10* gene regulates red color in apples, and its expression is higher in red-fleshed cultivars compared to white-fleshed ones, correlating with increased anthocyanin production during maturation [51]. **Overexpression** of the *MdMYB10* gene in apple plants enhanced pigmentation through increased anthocyanin levels and also resulted in a greater than 20-fold **increase** in total anthocyanins in whole fruit [25,52]. Espley et al. [1] reported that *MdMYB10* up-regulation directly boosted anthocyanin levels by regulating its biosynthetic pathway and indirectly enhanced flavonoid production through increased pathway flux. *MdMYB10* was associated with early ethylene production, possibly via ERF106, which accelerated fruit maturity and elevated polyphenol oxidase (PPO) and other peroxidase levels. These combined effects triggered early ripening, leading to the production of peroxidase enzymes and additional substrates, ultimately resulting in Internal Browning Fruit Disorder (IBFD).

**Fig. 3:**
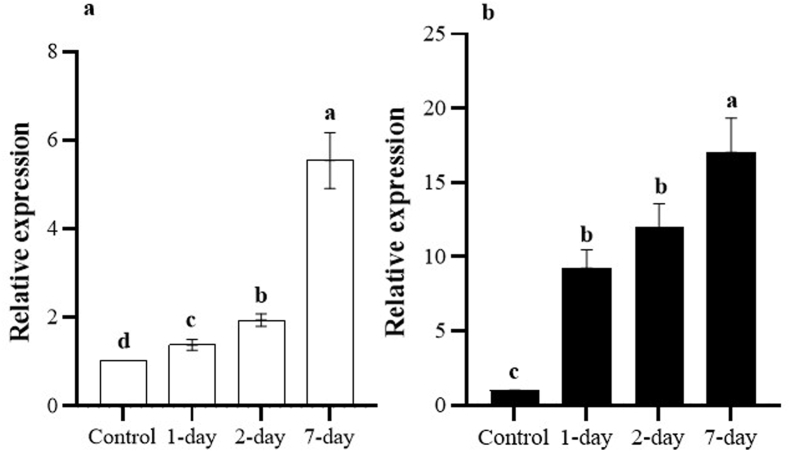
Relative expression of the *MdMYB10* gene in fruits of two apple cultivar after 1, 2 and 7 days of *M. laxa* infection. Transcript level of each cultivar in control conditions (control) was used as the calibrator of each time points. a) Lebanese and b) Gala Imperial. The error bars indicate the standard error of the mean (SEM) and different letters show significant differences between columns.

**Fig. 4:**
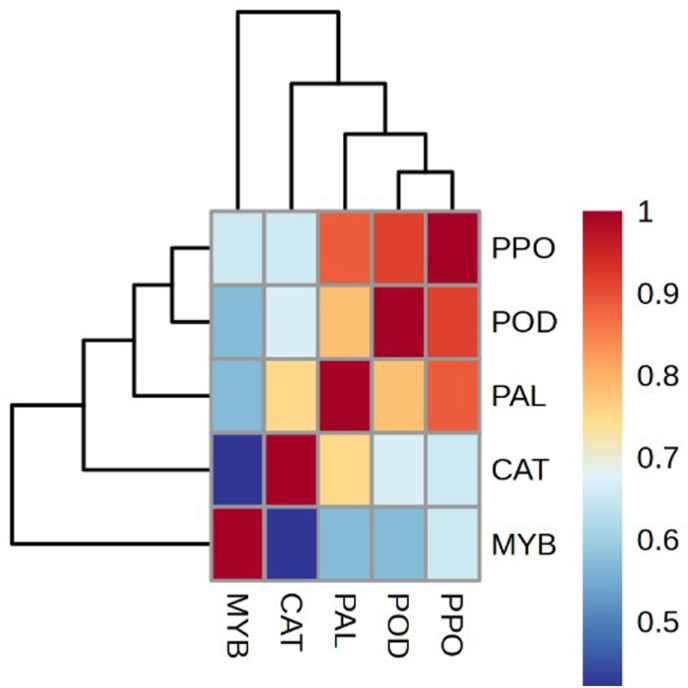
Heat map correlation among *MdMYB10* expression and enzymes' activities (POD, PPO, PAL, and CAT) in apples fruits after *M. laxa* infection.

Our results showed that *MdMYB10* expression increased significantly in both apple cultivars at all-time points after inoculation with *M. laxa* compared to the control. The highest level of upregulation in both cultivars was at 7 days after inoculation, and the transcript level of *MdMYB10* in Gala Imperial was **significantly higher** than in Lebanese (Fig. 3). Pearson's correlation coefficient was used to determine the relationship between *MdMYB10* expression and browning enzymes (Fig. 4). *MdMYB10* correlated with PAL, POD, and PPO by 0.49, 0.57, and 0.65, respectively. Moreover, POD and PPO showed the highest correlation (0.92), and PAL **was strongly correlated** with POD (0.78) and PPO (0.88). Overall, the results showed a strong positive correlation among the variables, particularly among POD, PPO, and PAL. Our results were in line with the study by Espley et al. [1], which reported that upregulating *MdMYB10* **led to an enhancement** of polyphenol oxidase (PPO) and peroxidative activities. Based on our results, it seems that *MdMYB10* is involved in the response to *M. laxa* infection. Moreover, upregulating *MdMYB10* **enhanced** PAL, PPO, and POD activities, which **activated** the defense mechanism in both cultivars, especially Gala Imperial, against *M. laxa* infection.

## 4 Conclusion

In the present study, the expression level of *MdMYB10* and enzyme activities (CAT, PAL, POD, and PPO) were assessed at 1, 2, and 7 days after inoculation by *M. laxa* in two apple cultivars (Imperial and Lebanese). The results revealed that all enzyme activities and *MdMYB10* expression significantly enhanced after infection, indicating their roles in the defense mechanism against brown rot disease in apples. Based on the trend of enzyme activities and *MdMYB10* expression, the positive correlation of *MdMYB10* upregulation with enhanced activities of PAL, POD, and PPO suggests that *MdMYB10* plays an important role in enhancing the activities of defense-related enzymes against *M. laxa*, especially in the Gala Imperial cultivar. Moreover, PAL activity peaked two days after inoculation, while POD and PPO reached their highest levels at 7 days after infection, suggesting that PAL is activated earlier due to its role in synthesizing phenolic compounds, which POD and PPO use as substrates in oxidation reactions. In addition, although PAL, POD, and PPO activities were enhanced after *M. laxa* inoculation, there were no significant differences in PPO and POD activities between cultivars, while a higher level of PAL activity was observed in Gala Imperial. Thus, this cultivar improves resistance to *M. laxa* infection by elevating PAL activity.

## CRediT authorship contribution statement

MRR: Conceptualization, Methodology. SS: Writing-original draft, Review & editing. FD and MK: Methodology. MF: Statistical analyses, Review & editing.

## Ethics approval and consent to participate

Not applicable.

## Availability of data and materials

No data was used to support the findings of this study.

## Human and animal rights

No animals/humans were used for studies that are the basis of this research.

## Funding

This research did not receive any specific grant from funding agencies in the public, commercial, or not-for-profit sectors.

## Declaration of competing interest

The authors declare that they have no known competing financial interests or personal relationships that could have appeared to influence the work reported in this paper.

## Data Availability

No data was used for the research described in the article.
